# Bacterial Symbiosis Maintenance in the Asexually Reproducing and Regenerating Flatworm *Paracatenula galateia*

**DOI:** 10.1371/journal.pone.0034709

**Published:** 2012-04-03

**Authors:** Ulrich Dirks, Harald R. Gruber-Vodicka, Nikolaus Leisch, Silvia Bulgheresi, Bernhard Egger, Peter Ladurner, Jörg A. Ott

**Affiliations:** 1 Department of Marine Biology, University of Vienna, Vienna, Austria; 2 Department of Genetics in Ecology, University of Vienna, Vienna, Austria; 3 Ultrastructural Research and Evolutionary Biology, Institute of Zoology, University of Innsbruck, Innsbruck, Austria; 4 Department of Genetics, Evolution and Environment, University College London, London, United Kingdom; Academia Sinica, Taiwan

## Abstract

Bacteriocytes set the stage for some of the most intimate interactions between animal and bacterial cells. In all bacteriocyte possessing systems studied so far, *de novo* formation of bacteriocytes occurs only once in the host development, at the time of symbiosis establishment. Here, we present the free-living symbiotic flatworm *Paracatenula galateia* and its intracellular, sulfur-oxidizing bacteria as a system with previously undescribed strategies of bacteriocyte formation and bacterial symbiont transmission. Using thymidine analogue S-phase labeling and immunohistochemistry, we show that all somatic cells in adult worms – including bacteriocytes – originate exclusively from aposymbiotic stem cells (neoblasts). The continued bacteriocyte formation from aposymbiotic stem cells in adult animals represents a previously undescribed strategy of symbiosis maintenance and makes *P. galateia* a unique system to study bacteriocyte differentiation and development. We also provide morphological and immunohistochemical evidence that *P. galateia* reproduces by asexual fragmentation and regeneration (paratomy) and, thereby, vertically transmits numerous symbiont-containing bacteriocytes to its asexual progeny. Our data support the earlier reported hypothesis that the symbiont population is subjected to reduced bottleneck effects. This would justify both the codiversification between *Paracatenula* hosts and their *Candidatus* Riegeria symbionts, and the slow evolutionary rates observed for several symbiont genes.

## Introduction

A major challenge in symbiosis research is to identify the mechanisms that allow symbiotic partners to selectively establish and maintain symbiotic associations. In the particular case of bacteria residing inside animal host cells – commonly called bacteriocytes [1], [2], [3] – this involves differentiation and maintenance of bacteriocytes during the symbiotic part of the life cycle (intragenerational transmission) as well as symbiont transmission between host generations. In the latter case the symbionts can either be acquired from an environmental symbiont population (horizontal transmission) or passed on from the parental generation (vertical transmission) (reviewed in [4]). The vertical transmission of symbionts can occur both during sexual [5] and asexual reproduction [6], [7]. If symbionts can be acquired both from the environment and the parental generation, the transmission is called ‘mixed’. Strict vertical transmission typically results in tight codiversification of the partners [8]. Moreover, it requires a high degree of integration of the symbiotic life style into the host developmental program. Intracellular symbionts are typically restricted to specialized tissues or organs such as for example the bacteriome in various insects, the trophosome of deep-sea tubeworms (Siboglinidae) or the gill filaments of lucinid bivalves [9], [10], [11], [12], [13]. Bacteriome, trophosome and gill filaments are functionally analogous tissues that mainly consist of symbiont-housing bacteriocytes. In these three systems, bacteriocyte *de novo* formation (differentiation of aposymbiotic cells and acquisition of symbionts; often called “infection”) happens during the embryonic/larval development of the animals [3], [9], [14], [15], [16]. Once established in the insects and tubeworms, the bacteriocytes themselves proliferate, whereas in the case of lucinids, mitotic bacteriocytes have not been detected [12], [17], [18], [19]. All these hosts have in common that neither symbiont acquisition nor *de novo* differentiation of bacteriocytes happens in the adults. This restriction of *de novo* bacteriocyte determination and formation to a given life cycle stage complicates the investigation of these essential processes. Only for some eukaryote/eukaryote intracellular symbioses, such as those between cnidarians and intracellular algae, life-long uptake and release of symbionts has been reported [20], [21], [22].

**Figure 1:**
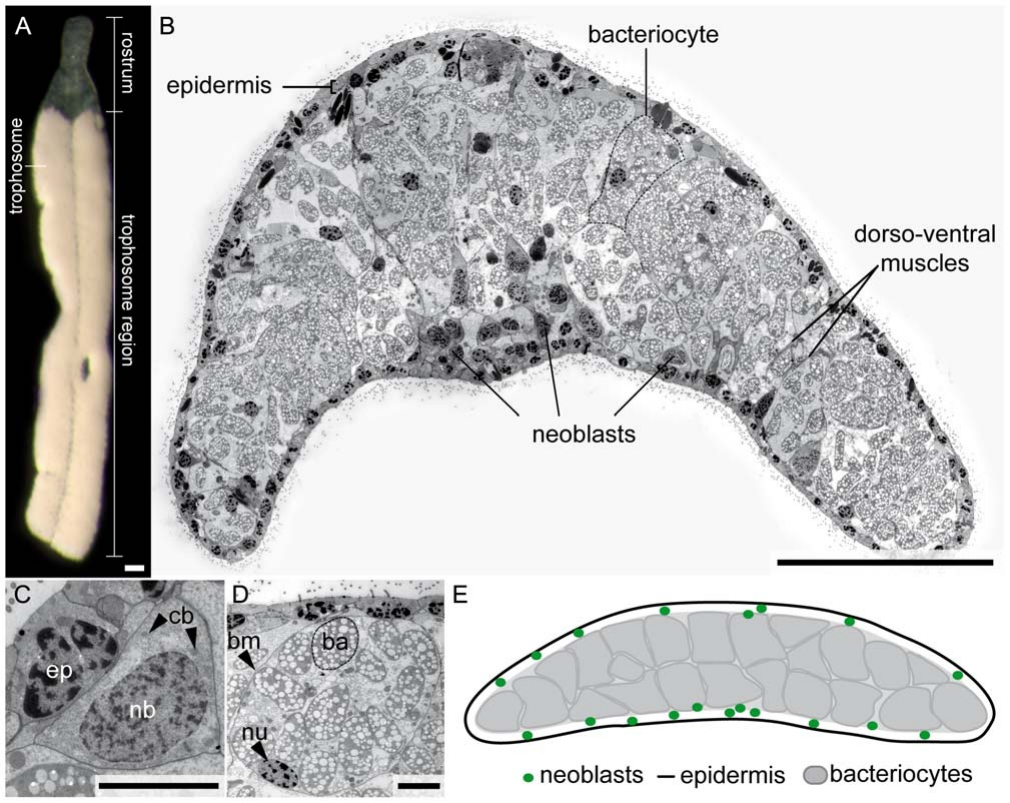
The Habitus of *P. galateia* and the Ultrastructure of the Trophosome Region. (A) Squeeze preparation of a live *P. galateia* specimen under incident light showing the smooth, silky appearance of the trophosome and the transparent rostrum. (B) TEM trophosome region cross section. One bacteriocyte (surrounded by a dashed line), the thin epidermis, neoblasts stem cells and dorso-ventral muscles are labeled. (C) TEM section of a neoblast (nb) proximal to the epidermis (ep); the cell contains highly active chromatin in the nucleus, chromatoid bodies (cb, arrowheads) and a high nucleo-cytoplasmatic ratio. (D) TEM section of a bacteriocyte with the delicate bacteriocyte membrane (bm, arrowhead); it contains the eukaryotic nucleus (nu, arrowhead) and numerous symbiotic bacteria (ba), which in turn contain bright refractive granules. (E) Schematic drawing of the trophosome region cross section reduced to the three types of cells and tissues most important for this study. Scale bars in (A) and (B) 50 µm, in (C) and (D) 5 µm.

The flatworm class Catenulida (phylum Platyhelminthes) are bilaterians with a simple bauplan [23]. Mouth- and gutless catenulids of the genus *Paracatenula* are members of the meiofauna in marine shallow-water sediments. They harbor intracellular chemosynthetic alphaproteobacterial symbionts, “*Candidatus* Riegeria”, which can equal the hosts' biomass [24], [25], [26]. The most intensively studied species of the genus, *Paracatenula galateia* Dirks et al., 2011, is dorso-ventrally flattened, about 300 µm wide and extremely variable in length (1–6 mm) (Figure 1A). The body consists of two parts: (1) the narrow and symbiont-free rostrum anterior to the brain and (2) the posterior “trophosome region”, which mainly consists of the symbiont-housing bacteriocytes (Figure 1A, 1D). Collectively, all bacteriocytes constitute a tissue termed “trophosome” in functional analogy to the trophosome of siboglinid tubeworms [10]. A recent study by Gruber-Vodicka, *et al*. [26] reports a tight codiversification between species of the genus *Paracatenula* and their specific symbionts possibly dating back up to 500 million years. This points to a strict vertical symbiont transmission in the genus, although the reproduction strategies were unknown at the time we started this study. Most catenulids, however, predominantly reproduce by a type of asexual fission termed paratomy, during which new clonal worms grow from the posterior end of the animal, thus generating a chain of zooids (the Latin word “catenula” means “small chain”). The zooids subsequently develop into separate individuals and detach from the mother zooid. Sexual reproduction is rarely observed in catenulids [27] and has never been observed in *Paracatenula* [25], [28].

Platyhelminthes possess pluripotent (maybe totipotent) stem cells – the so-called neoblasts – during their whole lifespan [29], [30], [31]. Neoblasts are self-renewing and the sole source of all differentiated cell types including the germ cells (reviewed in [32]). Morphologically, they are characterized by chromatoid bodies, a small size, a very high nucleo-cytoplasmatic ratio and a characteristic heterochromatin pattern [33], [34], [35], [36]. In addition to their function in cell renewal, neoblasts provide exceptional regenerative powers to many flatworm taxa (reviewed in [37], [38]).

In this study, we used pulse and pulse-chase incubations with thymidine analogues to label and trace S-phase cells in *P. galateia*. We show that bacteriocytes, along with all other somatic cell types, derive from aposymbiotic neoblast stem cells. Their continuous production allows bacterial symbiosis maintenance in an animal, which reproduces by asexual fragmentation.

## Materials and Methods

### Ethics Statement

All animal work has been conducted according to relevant national and international guidelines. All necessary permits were obtained for the described field studies. Permission for the export of invertebrate animals from Belize (Central America) was issued by the Ministry of Agriculture and Fisheries of Belize. The permission holder for the collection of invertebrates in Egypt is the Dahab Marine Research Center (DMRC), which hosted us during our fieldwork. No animals were exported from Egypt.

### Sampling

Since none of the *Paracatenula* species can be grown in culture, all experiments requiring live animals were performed immediately after sampling in the field laboratories of Carrie Bow Cay, Belize (16°48′11″N, 88°04′55″W) and Dahab, Egypt (28°28′13.83″N, 34°30′32.51″E). Sediments were collected in shallow water near Carrie Bow Cay (March 2009 and October 2010) or in the “Napoleon Reef” in Dahab (June 2010). The worms were extracted by gently shaking the sand with ample amounts of filtered sea water (FSW) (0.45 µm filter) followed by pouring the supernatant through a 63 µm pore-size mesh that retains the animals. Animals were then immediately and very carefully washed from the mesh into petri dishes and picked by hand with *Pasteur* pipettes under a dissecting microscope. Then they were either fixed immediately (see below) or kept alive for different experiments for up to 16 days in 2 ml glass vials containing FSW and a small amount of sediment from the sampling area.

### Transmission Electron Microscopy (TEM)

Freshly collected animals were immediately relaxed in a MgCl_2_ solution isotonic to SW for 5 min and fixed in 2.5% glutaraldehyde in a 0.1 M sodium cacodylate buffer, post fixed for 2 h with 1% osmium tetroxide in 0.1 M sodium cacodylate buffer (pH 7.3) and, after dehydration, embedded in Low Viscosity Resin (Agar Scientific, England). Complete ultra-thin cross-sections mounted on formvar-coated slot grids were post stained with 0.5% uranyl acetate for 20 min and lead citrate for 6 min. Sections were viewed on a Zeiss EM-902 (Zeiss, Oberkochen, Germany) and images were recorded with an Olympus (Tokyo, Japan) SharpEye camera system using the AnalySIS 5.0 software (Zeiss, Oberkochen, Germany). Single images were merged and further processed with the Adobe Photoshop CS5 software (Adobe Systems complex, San Jose, California, U.S.A.).

### Thymidine Analogue and Nocodazole Incubations

For labeling of S-phase cells the thymidine analogues EdU (5-ethynyl-2′-deoxyuridine, Click-it EdU Kit) (Invitrogen Life Technologies, Carlsbad, California, U.S.A.) or BrdU (5′-bromo-2′-deoxyuridine, Sigma-Aldrich, St. Louis, Missouri, U.S.A.) were dissolved in FSW to a concentration of 2.5 mM. Animals were incubated for the length of the labeling pulse (always 30 min) in one of these solutions. Each pulse was followed by five washes in FSW and eventually a chase in FSW (ranging from 12 h up to 148 h, n≥5 for each chase). Incubations (including chase incubations) in FSW, containing 1 µg/ml nocodazole (Sigma-Aldrich, St. Louis, Missouri, U.S.A.) for 12 h, were used to arrest cells in mitosis. Afterwards the animals were immediately relaxed in MgCl_2_ solution isotonic to SW for 5 min followed by fixation in 4% formaldehyde in phosphate buffered saline (PBS) for 12 h at 4°C. Fixed animals were stored in PBS for up to three weeks at 4°C or in pure methanol at −20°C for longer periods.

### Click Chemistry and Immunocytochemistry

Alexa fluor 488-azide fluorescent dye (Invitrogen Life Technologies, Carlsbad, California, U.S.A.) was covalently connected to the EdU-label in fixed specimens of *P. galateia*, performing the “click reaction” following the protocol of the EdU click-iT Kit. Stainings with antibodies against BrdU (Becton & Dickinson, Franklin Lakes, New Jersey, U.S.A.), serotonin (staining serotonergic nerves) (Sigma-Aldrich, St. Louis, Missouri, U.S.A.) and phosphorylated histone 3 (staining mitotic cells) (Millipore, Billerica, Massachusetts, U.S.A.) were performed according to the immunostaining protocols established by Ladurner *et al*. [31], [39], except for the protease treatment. We used Proteinase K (Sigma-Aldrich, St. Louis, Missouri, U.S.A.) at a final concentration of 0.1 mg/ml for up to 10 min at room temperature. Fluorescently stained whole animals were mounted on slides and scanned with a confocal laser-scanning microscope Zeiss LSM 510 (Zeiss, Oberkochen, Germany).

For permanent non-fluorescent labeling, we used a horseradish peroxidase (HRP) catalyzed deposition staining approach (see [40]) except that we used secondary antibodies directly conjugated to HRP (GE Healthcare, Fairfield, Connecticut, U.S.). Pre-stained whole animals were embedded in LR-white resin (London Resin Company, London, England), and 2-µm-thin cross sections of the trophosome region were produced and counterstained with 1% methylene blue. The sections were examined under the bright field microscope Zeiss Axio Imager.

Images were further analyzed and processed with the Zeiss LSM Image Browser and the Adobe Photoshop CS5 software. Illustrations were produced with Adobe Illustrator CS5 software (Adobe Systems complex, San Jose, California, U.S.A.).

For both EdU-click-iT and antibody stainings, negative controls were conducted.

### Rostrum Amputation

Freshly collected *P. galateia* and *P.* cf. *polyhymnia* were reversibly anesthetized with MgCl_2_ solution isotonic to FSW (see above). Using a clean razor blade the worms (n = 50 for each species) were cut transversally posterior to the brain region or further posterior in the trophosome region. Fragments were kept in glass vials as described above and regeneration was observed and photographically documented in 12 h intervals using a dissecting microscope with a camera.

## Results

### Tissue Composition of the Trophosome Region

**Figure 2:**
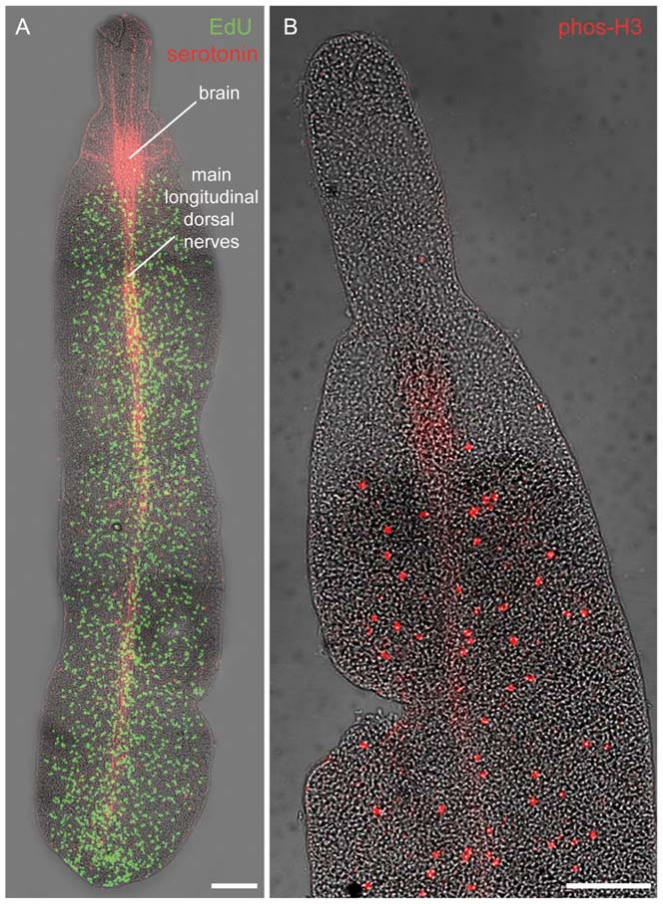
Distribution of S-phase and Mitotic Cells. (A) Confocal fluorescence projections of 30 min EdU pulse-labeled S-phase cells (green) and serotonergic nerves (red) superimposed with interference-contrast images of *P. galateia* whole-mounts. S-phase cells are restricted to the body region posterior to the brain. Longitudinal dorsal nerves extend from the brain to the posterior end of the body. (B) Superimposition of interference-contrast and confocal fluorescence projections of mitotic cells (red) in the anterior body region of a 12 h nocodazole-treated *P. galateia*. Mitotic cells are restricted to the body region posterior to the brain. Scale bars in (A) and (B) 100 µm.

**Figure 3:**
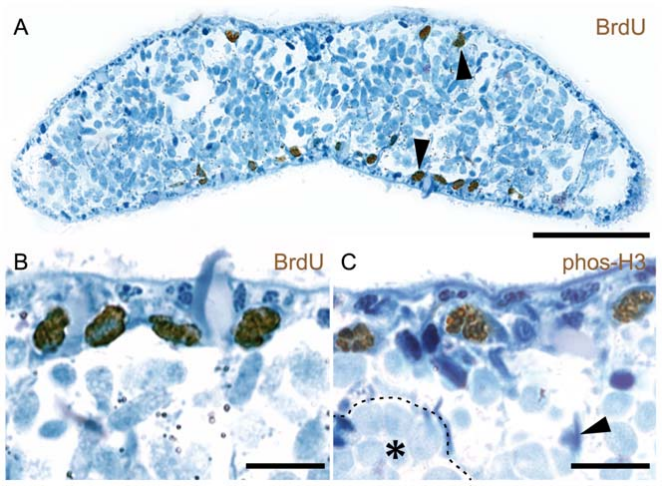
S-phase and Mitotic Cells Are Found in Subepidermal Positions. Bright field micrographs of immuno-stained trophosome region sections. All sections were counterstained with methylene blue (blue). (A) 30 min BrdU pulse-labeled S-phase cells (brown) in subepidermal positions on the dorsal and ventral side of the trophosome region (arrows). (B) Detailed micrograph of BrdU-labeled cells in sub-epidermal positions. (C) Detailed micrograph of mitotic cells (brown) in sub-epidermal positions of trophosome region cross sections. Mitotic cells were accumulated by 12 h nocodazole treatment. One bacteriocyte is indicated with an asterisk and surrounded by a dashed line. A nucleus presumably belonging to a muscle cell is labeled by an arrowhead. Scale bars in (A) 50 µm, (B) and (C) 10 µm.

We analyzed the tissue composition of the trophosome region using TEM cross sections of *P. galateia*. Three prominent cell and tissue types were identified: (1) a thin epidermis (Figure 1B), (2) neoblast stem cells (Figure 1B, 1C) and (3) the trophosome consisting of large bacteriocytes (Figure 1B, 1D and S1 showing a dividing symbiont). Rarely, other differentiated cell types such as dorso-ventral muscles permeated the trophosome between the bacteriocytes (Figure 1B). The neoblasts were identified based on their characteristic pattern of heterochromatin, the high nucleo-cytoplasmatic ratio and the presence of chromatoid bodies in the cytoplasm (Figure 1C). They were mostly found proximal and adjacent to the epidermis of the dorsal and ventral side. A schematic trophosome cross section is shown in Figure 1E.

### Neoblasts Are Restricted to the Trophosome Region

We determined which cell types are proliferating in *P. galateia* by using thymidine analogue pulse-labeling. In whole-mounts of specimens pulse-labeled with EdU (or BrdU) (30 min pulse length) we detected S-phase cells exclusively in the posterior body region (trophosome region) (n = 10). In EdU-labeled specimens in which serotonergic nerves were also stained, the S-phase cells were evenly distributed posterior to the brain (Figure 2A). On semithin sections of the trophosome region of pulse-labeled specimens, we found the labeled cells proximal to the epidermis on both the dorsal and ventral side (n = 30 from three specimens) (Figure 3A, B). This neoblast distribution is consistent with that described on TEM sections (above). No label was observed in already differentiated cells types such as epidermal or muscle cells, in nuclei of bacteriocytes or in the bacterial symbionts themselves.

The number of mitotic cells, which were detected with an antibody against phosphorylated histone 3, was always low and highly variable between different individuals. We used nocodazole (an inhibitor of microtubule polymerization) to arrest and accumulate cells in mitosis. In whole-mounts of nocodazole-treated worms, mitotic cells were evenly distributed in the trophosome region but absent in the rostrum (n = 10) (Figure 2B). This mirrors the distribution of the S-phase neoblasts (Figure 2A). On stained sections we always found mitotic cells proximal to the epidermal cells, the same position described for S-phase neoblasts (n = 30 from three specimens). We did not find any mitotic cells in the epidermis or inside the trophosome. In whole-mounts of EdU-pulse-labeled worms subjected to a 12 h nocodazole chase, most of the mitosis label overlapped with the EdU signals (Figure S2). This indicates that mitotic cells are derived from previously labeled S-phase cells.

### Neoblasts Are the Source of Bacteriocytes and All Other Somatic Cell Types

**Figure 4:**
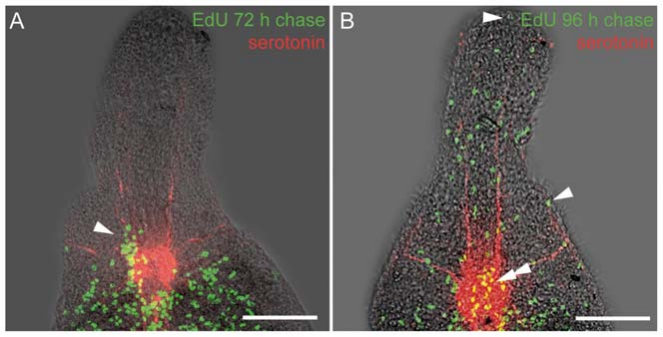
Pulse-Chase Labeled Cells Migrate into the Rostrum. Confocal projections of EdU pulse-chase labeled cells (green) and serotonergic nerves (red) superimposed with interference-contrast images of *P. galateia* whole-mounts. Only the anterior parts of the worms are shown. (A) Worms with EdU-pulse and 72 h-chase showing labeled cells in the moment of entering the rostrum (arrowhead). (B) After 96 h, chase-labeled cells are distributed over the whole length of the rostrum. Label is visible in the epidermis and in nerve cells of the brain (yellow double label, arrows). Scale bars in (A) and (B) 100 µm.

**Figure 5:**
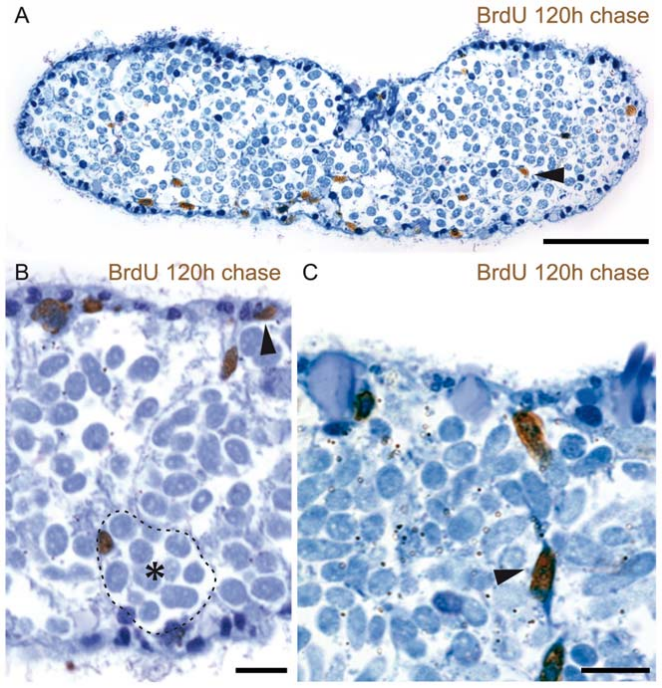
Pulse-Chase Labeled S-phase Cells in Trophosome Region Cross Sections. Bright field micrographs of 30 min BrdU pulse and 120 h chase immuno-labeled *P. galateia* trophosome region cross sections. All sections were counterstained with methylene blue (blue). (A) BrdU-labeled cells (brown) were distributed in all regions of the section including the trophosome (arrowhead). (B) Detailed micrograph of BrdU-labeled bacteriocyte (asterisk, surrounded by a dashed line) and labeled epidermal cells (arrowhead). (C) Other BrdU-labeled cell types between the bacteriocytes. Scale bars in (A) 50 µm, (B) and (C) 10 µm.

To study cell migration and differentiation into bacteriocytes and other cells types, we performed EdU and BrdU pulse-chase incubations. Pulse-labeled worms were subjected to increasing chase length to determine when cell migration and differentiation starts. The first cell migrations were detected after 72 h chase, when three out of five specimens showed clusters of EdU-labeled cells entering the rostrum (arrowhead in Figure 4A). After 96 h chase, all specimens showed labeled cells distributed over the whole length of the rostrum (n = 5); this indicates ongoing migration and differentiation. In these whole-mounts, we identified labeled cells in the epidermis (arrowhead in Figure 4B) and in the brain (double arrowhead in Figure 4B). In semithin sections of specimens with BrdU pulse and 120 h chase (n = 60 from three specimens) and 148 h chase (n = 40 from two specimens), we observed labeled cells in the epidermis, in subepidermal positions and in the trophosome (Figure 5A and B). In the trophosome, nuclei of bacteriocytes were labeled (dotted line in Figure 5B), as were those of cells between bacteriocytes which probably belong to dorsoventral muscles, nerves or mesenchymal cells (Figure 5C). No label was detected in the symbionts.

### *P. galateia* Can Reproduce by Paratomy and Can Regenerate From Small Fragments

**Figure 6:**
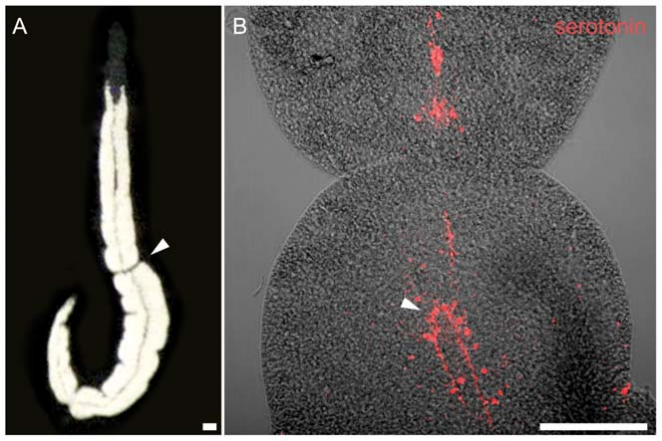
Nerve Rearrangement in a Fission Plane Area. (A) *In vivo* squeeze preparation of *P. galateia* under incident light. The animal exhibits a fission plane (arrowhead) in the trophosome region. (B) Confocal fluorescent projection of a serotonin staining (red) superimposed with an interference-contrast image of a fission plane area. The longitudinal nerves (red) are separated into an anterior and posterior fraction. A commissure (arrowhead) in the posterior fraction indicates the reorganization of the nerves. Scale bars in (A) and (B) 100 µm.

Fission planes (FPs) are constrictions perpendicular to the anterior-posterior axis correlated with asexual fission in flatworms. FPs were abundant in freshly collected *P. galateia* and other species of the genus (arrowheads in Figure 6A and S3). They were most common in long specimens (≥3 mm) and were confined to the trophosome region. The main longitudinal serotonergic nerves in specimens with FPs and stained with anti-serotonin antiserum were interrupted and well separated into nerve fractions anterior and posterior to the FP (Figure 6B). A prominent commissure in the posterior nerve section shows the reorganization of the central nervous system before fragment separation (arrowhead in Figure 6B). This indicates that the FPs are not due to mechanical stress but are signs of paratomy (transverse fission during which differentiation of new organs occurs prior to separation from the parental animal; see introduction). Under culture conditions, however, FPs did not progress and no detachment of fragments could be observed within the limited observation period of 16 days.

**Figure 7:**
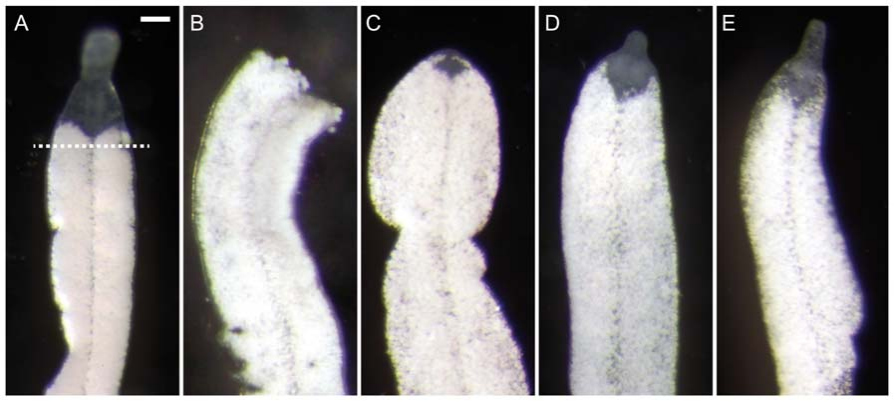
Trophosome Region Fragments Can Regenerate the Rostrum. Squeeze preparations of live *P. galateia* under incident light. (A) Intact *P. galateia* prior to rostrum amputation. The dashed line indicates the level of cutting. (B) *P. galateia* directly after rostrum amputation (stage 0). Small shreds of injured bacteriocytes leak out the wound. (C) Regeneration stage 1: the wound is closed and a symbiont-free area on the anterior tip indicates ongoing rostrum regeneration. (D) Regeneration stage 2: a small outgrowing rostrum is visible on the anterior tip. (E) Regeneration stage 3: the new rostrum has its final characteristic shape and has grown almost to its full size. Scale bars in (A–E) 100 µm.

To investigate the fate of headless *P. galateia* trophosome region fragments, which are probably produced in the natural environment, we tested their ability to regenerate a rostrum. Intact worms were decapitated posterior to the brain (dashed line in Figure 7A). Subsequently, four stages of the rostrum regeneration process were observed. Decapitation resulted in a trophosome region fragment of varying length (≥1 mm) with a wound at its anterior end (stage 0) (Figure 7B). Within the following two days the wound surface was minimized and closed before a symbiont-free anterior region appeared (stage 1) (Figure 7C). This was followed by the outgrowth of the rostrum tip (stage 2) (Figure 7D), leading to the formation of the typical shoulder-shaped transition between rostrum and trophosome region (stage 3) (Figure 7E). The time to complete rostrum regeneration varied between 12 and 16 days (n = 10 for each stage). In addition, rostrum regeneration after decapitation was studied in another species, *P.* cf. *polyhymnia*, with comparable results (Figure S4) except that rostrum regeneration was completed within only 48–72 h. We also cut *P. galateia* with long trophosome regions twice or three times. These fragments (length ≥0.5 mm) were also capable of rostrum regeneration, regardless whether they were cut on only one or both sides (n = 20) (Figure S5).

In the rostrum fragments resulting from the decapitation, the wound also closed within the first days. Although all of these fragments retained a small piece of trophosome after cutting (Figure 7A, dashed line indicates the cutting plane) no regrowth occurred (n≥40), probably due to the small number of neoblasts remaining in such pieces (compare Figure 2A). All rostrum fragments died within the 16 days of observation.

To determine how many symbionts can be transmitted to the asexual offspring during paratomy or inside accidently produced fragments, we estimated the number of bacterial cells in a 1 mm long (A–P axis) slice of the trophosome region based on the mean dimensions of their cells and their number on cross sections (15 sections from 3 specimens). Our estimates suggest that 36,000–42,000 symbiont cells are transmitted per millimeter trophosome region upon each fragmentation event.

## Discussion

### The Bacterial Symbionts Are Integrated into the Ancient Cell-Renewal Machinery of the Flatworm Host

Platyhelminthes are exceptional model systems in stem cell and regenerative biology [41]. Their simple neoblast stem cell system, in which all differentiated cells constantly derive from undetermined stem cells, provides a unique opportunity to investigate the development of specific cell types [32]. In this study we identified neoblast stem cells as the source of bacteriocytes and other somatic cell types in *Paracatenula*. With a combined approach using ultrastructure, incubations with thymidine analogues and immunostaining, we provide several lines of evidence for this: (1) the detection of cells with typical flatworm-neoblast features (see introduction) in *P. galateia;* (2) the restriction of neoblasts (S-phase and mitotic cells) to the posterior body region, which is comparable with that found in many other flatworm species [29], [31], [42]; (3) migration of pulse-chase labeled S-phase cells and their differentiation into various cell types known from many other flatworm models [29], [31], [41]. All these results show that aposymbiotic neoblasts of adult *P. galateia* continuously generate bacteriocytes and all other somatic cell types.

Although various cnidarians including hard corals are known to be capable to exchange their intracellular algae with the environment throughout their life cycle, in all reported intracellular animal/bacteria associations *de novo* bacteriocyte formation and symbiont acquisition happens only once [3], [9], [15], [16]. Adult siboglinid tubeworms (*Lamellibrachia luymesi* and *Riftia pachyptila*) possess unipotent bacteriocyte stem cells that proliferate and produce new bacteriocytes. During their division the symbionts are transferred to both daughter cells. One daughter retains the status of an unipotent stem cell, the other terminally differentiates and loses the capability to divide [17]. This is in agreement with the general mode of tissue renewal in annelids, whose needs are met by proliferating multi- and unipotent tissue-specific stem cells [43], [44]. In the aphids *Acyrthosiphon pisum* and *Megoura viciae* as well as in the cockroach *Periplaneta americana de novo* bacteriocyte formation from aposymbiotic cells is restricted to early developmental stages. During postembryonic development the bacteriocytes themselves proliferate [3], [12], [14], [18], [45]. In adult aphids, however, they stop dividing and the stock of symbionts is progressively reduced due to repeated vertical symbiont transfer to the offspring. The post-reproductive aphids are free of symbionts [12]. All the presented modes of bacteriocyte production obey the central cell renewal strategy of insect development: totipotency is restricted to germ cells, and all other stem cell types become progressively restricted from pluripotent (during embryonic development) to tissue-specific multipotent or unipotent stem cells (adults) (reviewed in [46]). The larvae of lucinid bivalves get infected by specific environmental bacteria. This infection can artificially be delayed or prevented when keeping the animals in aseptic sediment. Postembryonic aposymbiotic lucinids, however, remain receptive for symbionts and become infected as soon they get in contact [19]. After the initial infection, source and fate of bacteriocytes in adults are not known. Mitotic bacteriocytes have not been detected yet [2], [13], [19]. We have shown here that the remarkable neoblast stem cell system enables adult *P. galateia* to produce bacteriocytes *de novo* at any time of the life span. Therefore *P. galateia* is a promising system to study bacteriocyte determination and differentiation.

All the animal hosts discussed above have integrated bacteriocyte production into their conventional cell renewal systems. In general, cell renewal strategies of an animal taxon should be indicative for bacteriocyte formation and maintenance in their respective symbiotic tissues. Also acoelomorph flatworms, whose phylogenetic placement is highly disputed [47], [48], [49], [50], have a totipotent stem cell system with comparable features [49], [51], [52]. It would therefore be interesting to study the formation process of the symbiont-housing cells in the acoels *Symsagittifera roscoffensis* (formerly *Convoluta roscoffensis*) Graff 1891 [53], [54] or *Convolutriloba longifissura* Bartolomaeus & Balzer 1997 [55], [56].

We could not clarify how symbionts enter newly formed *P. galateia* pre-bacteriocytes (cells determined to become bacteriocytes). There are several possibilities: (1) bacteriocytes release symbionts to the extracellular space; these are then phagocytized by pre-bacteriocytes, (2) bacteriocytes transiently fuse with the pre-bacteriocytes, forming a syncytium, and transfer symbionts, or (3) bacteriocytes pinch off symbiont-containing vesicles which subsequently fuse with pre-bacteriocytes. Inside the bacteriocytes each symbiont is encapsulated by a host-derived membrane [24]. This should simplify symbiont transfer across the hosts' cell membranes. An uptake of new bacteria from the environment is highly unlikely since the worm has no mouth and gut and we never found any indication for phagocytosis of particles via the skin.

We never detected BrdU or EdU incorporation into the DNA of the symbionts, although they apparently proliferate in the bacteriocytes (Figure S1). This is consistent with other studies [17], [57]. Fuhrman & Azam [58] found evidence that some autotrophic bacteria do not incorporate thymidine analogues. Since the alphaproteobacterial *Paracatenula*-symbionts are chemoautotrophic, this might be a possible explanation [26].

### *P. galateia* Reproduces Asexually and Regenerates

Many catenulids, such as members of the genera *Catenula* and *Stenostomum*, reproduce predominantly by paratomy [27], [42], [59]. The same genera also exhibit high regenerative powers. Our results indicate that *P. galateia*, *P.* cf. *polyhymnia* and probably all other species of the genus (own unpublished observation) are capable of paratomy and can regenerate the rostrum. Regeneration of the trophosome from rostrum fragments was, however, not observed. Possible explanations may be the low number of neoblasts in the rostrum piece and/or insufficient nutrition due to the few bacteriocytes in the rostrum fragment when cut right posterior to the brain.

**Figure 8:**
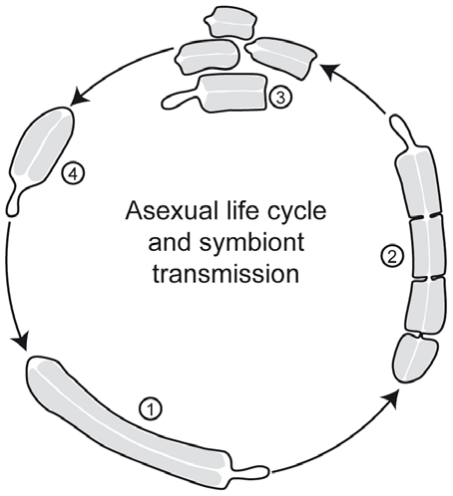
Scheme of the Hypothetical Asexual Reproduction by Paratomy. Drawing of *P. galateia* reproducing by paratomy (grey = trophosome). Paratomy starts when the trophosome extends to a certain length (1). Progressively, constrictions appear perpendicular to the anterior-posterior axis and zooids develop (2) which finally detach from the mother zooid (3). Then, each of the zooids grows to a complete worm (4), whereas the trophosome region continuously grows until it extends to a certain size and the process of paratomy starts again.

Natural disturbances such as wave action and bioturbation by macrofauna may cause accidental fragmentation in these fragile animals [25]. The frequent finding of fragments or battered and partly regenerated individuals in our field collections suggests the importance of regeneration in *Paracatenula's* natural environment. Amputation experiments demonstrated the potential of different *Paracatenula* species to regrow the rostrum even in small body fragments. Under good growth conditions, repeated paratomy and fragmentation/regeneration can lead to an exponential growth of the population, helping to explain the extremely patchy distribution of the worms (own unpublished observation). Figure 8 schematically outlines the hypothetical reproduction of *Paracatenula* by paratomy. Since sexual reproduction has never been observed in *Paracatenula* species, asexual fragmentation is probably the predominant mode of reproduction [25], [28].

### Asexual Transmission of Intracellular Symbionts

The diversity of symbiont transmission strategies between host generations were recently reviewed [4], [60]. Horizontal and vertical transmission are the two fundamentally different modes described (see introduction). Trophosome region fragments of *P. galateia* always contain many hundreds to thousands of bacteriocytes. As a consequence the worm vertically transmits high numbers of symbionts (36,000–42,000 per mm trophosome region) to the asexual offspring. This vertical “bacteriocyte transmission” results in a permanent host-symbiont liaison during asexual reproduction. We have no evidence for sexual reproduction in the genus *Paracatenula* [25], [28], but even if this would occur, horizontal transmission is unlikely given the tight codiversification reported for hosts and symbionts [26]. In most other systems, intracellular symbionts have to undergo a complex journey during vertical transmission. They must translocate from the parental symbiont-housing tissue into either germ cells or the developing progeny. In both cases the symbionts experience environmental changes, especially if they have to pass an extracellular space and risk losing contact with the host (reviewed in [4]). Asexual reproduction by fragmentation or budding is common in other symbiotic metazoans such as corals. These may asexually transmit their intracellular algae, however, the newly formed polyps are not released into the sea, but usually stay in contact with the colony that generated them [61], [62]. The transmission of whole symbiont-housing cells to separate new individuals has been described for *Hydra viridis* and *Convolutriloba longifissura*. During budding of *H. viridis* the algal and bacterial symbionts are transmitted directly through dividing gastrodermal cells to the developing bud. Sexually produced offspring, however, have to take up both kinds of symbionts from the environment, showing that this intracellular association is not uninterrupted in time [22]. Accordingly, no codiversification can be observed between this group of hydroids and their symbionts. *C. longifissura* and *Waminoa brickneri* also transmit their intracellular algal symbionts during asexual fission, but a detailed investigation is pending [55], [63].

The strict vertical transmission of a limited number of symbionts to the offspring – the transmission bottleneck – bears the risk of accumulating slightly deleterious mutations in the symbiont pool through genetic drift [64], [65], [66], [67]. Buchner observed that fewer than 200 bacteria are transmitted to each offspring of the louse *Pediculus* [68]. In various aphid-species the number of symbiont cells transmitted ranged from 800–8000 [64]. In contrast, when *P. galateia* reproduces by paratomy or accidental fragmentation, the high number of transmitted symbionts minimizes bottlenecking.

In conclusion, the capacity of the *P. galateia*'s cell renewal machinery to produce bacteriocytes at any time of the life cycle will promote the understanding of a *dark side* of symbiosis research: bacteriocyte determination and differentiation. Moreover, this ancient two partner association is a promising system to study an undescribed symbiont transmission strategy which enabled a tight codiversification between *Paracatenula*-species and their *Candidatus* Riegeria symbionts.

## Supporting Information

Figure S1 **The Symbionts Proliferate Inside the Bacteriocytes.** Detailed TEM-micrograph of a dividing bacterial symbiont. Sulfur storage granules (white inclusions) visible in bacterial cells.
(TIF)

Figure S2 **The Pulse-labeled Cells Enter Mitosis After a Certain Chase Time.** Confocal fluorescence projections of EdU-labeled S-phase cells (green) and mitotic cells (red) in the *P. galateia* trophosome region. The worm was subjected to a 30 min EdU pulse followed by a 12 h nocodazole chase. (A) Double label of EdU and mitosis. All mitotic cells also show EdU S-phase label (yellow). (B) Same image section showing only the red mitosis label. Scale bar in (A) and (B) 10 µm.
(TIF)

Figure S3 **Fission Plane in the Trophosome Region of** ***P.*** **cf** ***polyhymnia.*** In vivo squeeze preparation of *P.* cf *polyhymnia* under incident light. (A) The animal exhibits a fission plane (arrowhead) in the trophosome region. The inset shows a higher magnification of the fission plane. Scale bar in the main figure 100 µm and in the inset 50 µm.
(TIF)

Figure S4 **Regeneration After Rostrum Amputation of** ***P.*** **cf** ***polyhymnia.*** Micrographs of regenerating *P.* cf *polyhymnia.* Live worms before (A) and directly after rostrum amputation (B). Rostrum regeneration 24 h (C) and 48 h (D) after amputation. Scale bar in (A–D) 50 µm.
(TIF)

Figure S5 **Regeneration of Tiny Trophosome Region Fragments of** ***P. galateia.*** Micrograph of a 0.5-mm-long regenerating trophosome region fragment 14 days after rostrum amputation. A small rostrum is visible on one side (arrowhead). Scale bar 100 µm.
(TIF)
