# Supplementary figures and images for: Bacterial Symbiosis Maintenance in the Asexually Reproducing and Regenerating Flatworm Paracatenula galateia
Source: PLoS One. 2012 Apr 3;7(4):e34709. doi: 10.1371/journal.pone.0034709 (PMC3317999; doi:10.1371/journal.pone.0034709)

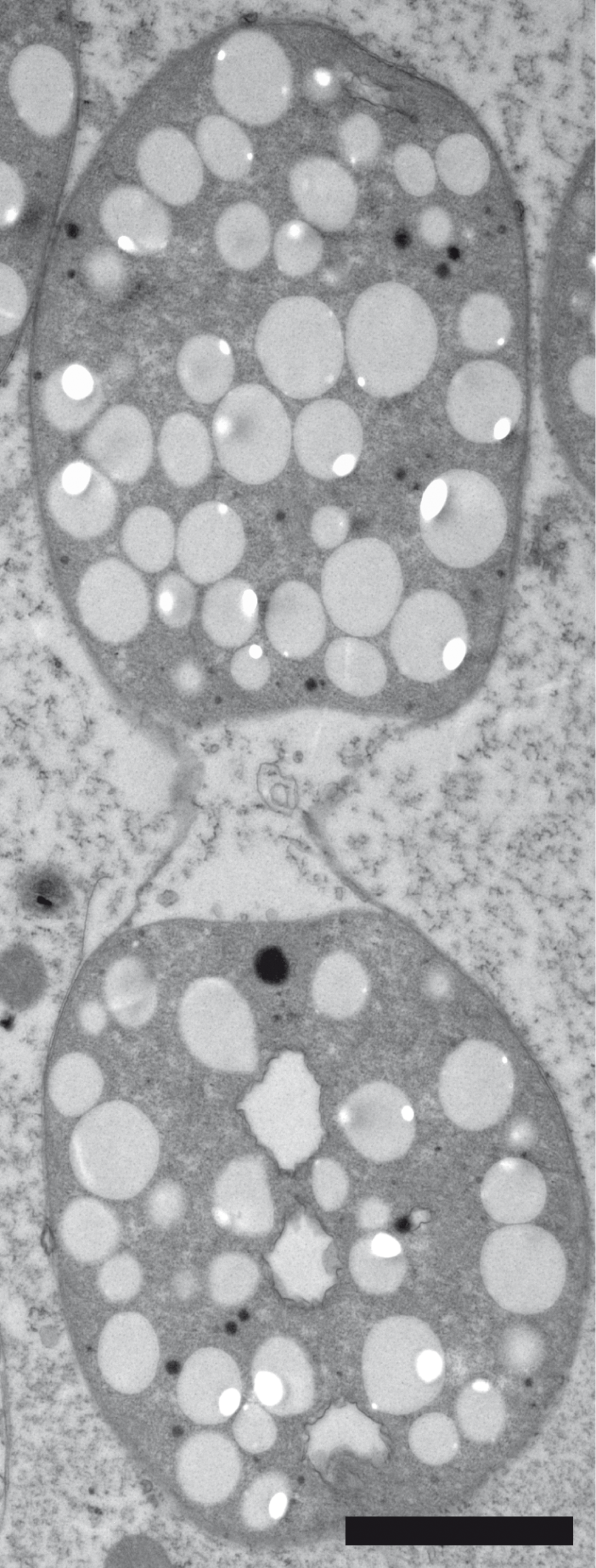

Supplement: Figure S1 — The Symbionts Proliferate Inside the Bacteriocytes. Detailed TEM-micrograph of a dividing bacterial symbiont. Sulfur storage granules (white inclusions) visible in bacterial cells. (TIF) [file pone.0034709.s001.tif]

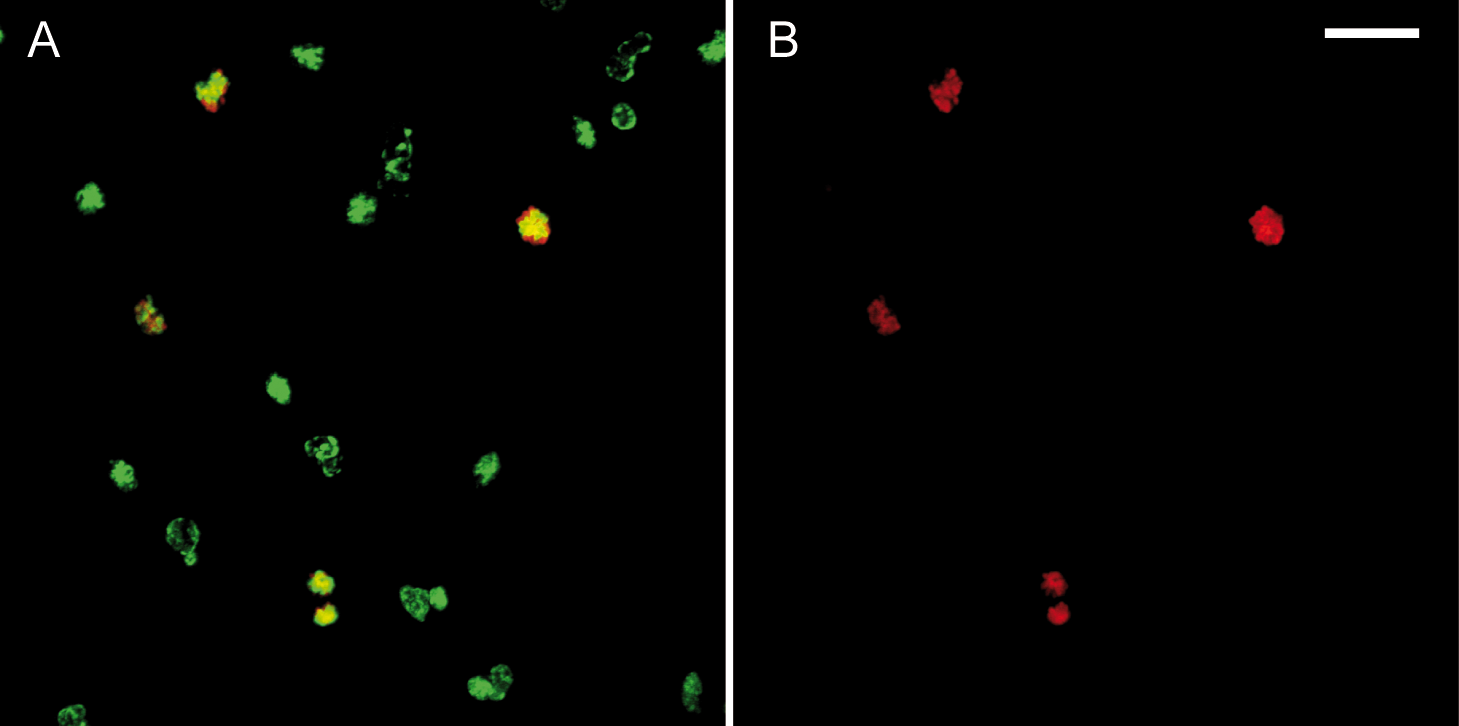

Supplement: Figure S2 — The Pulse-labeled Cells Enter Mitosis After a Certain Chase Time. Confocal fluorescence projections of EdU-labeled S-phase cells (green) and mitotic cells (red) in the P. galateia trophosome region. The worm was subjected to a 30 min EdU pulse followed by a 12 h nocodazole chase. (A) Double label of EdU and mitosis. All mitotic cells also show EdU S-phase label (yellow). (B) Same image section showing only the red mitosis label. Scale bar in (A) and (B) 10 µm. (TIF) [file pone.0034709.s002.tif]

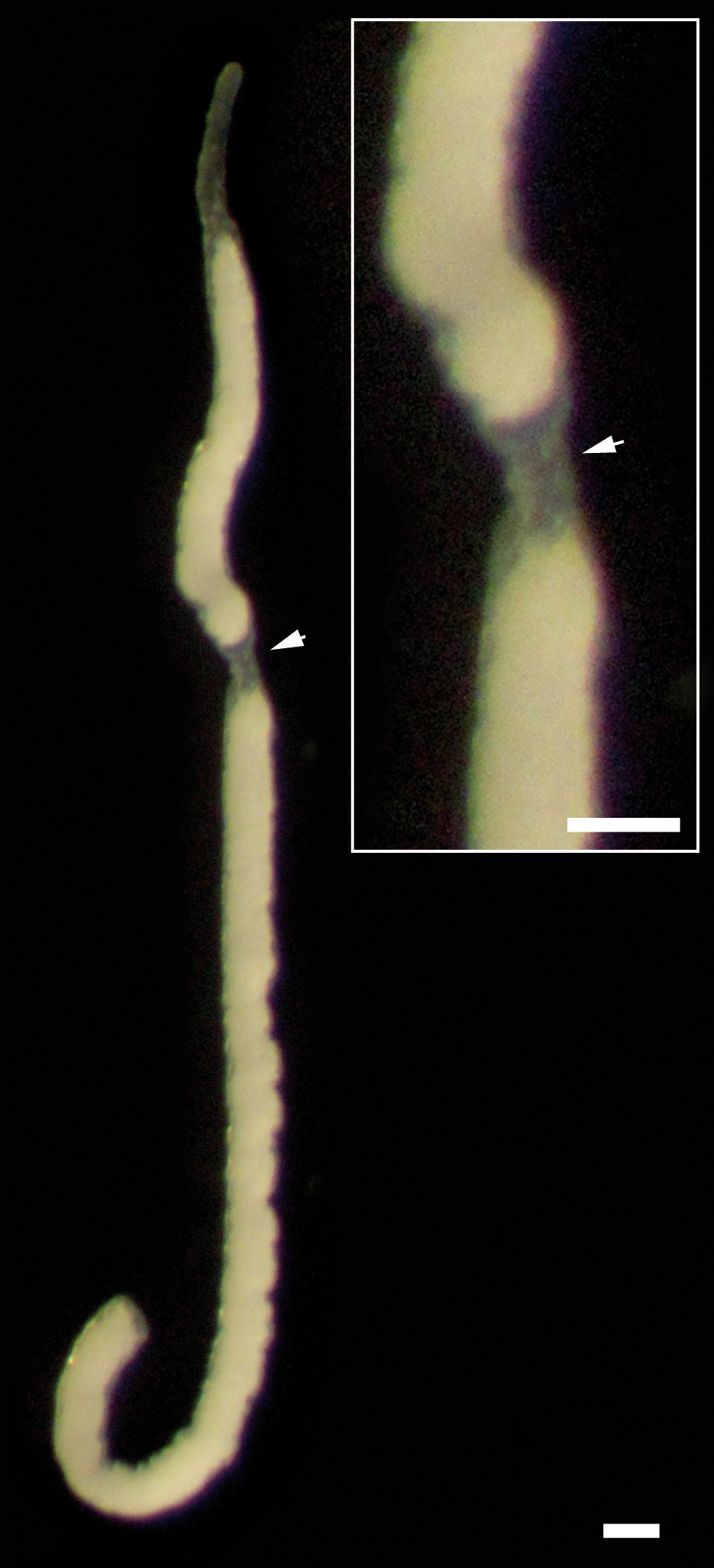

Supplement: Figure S3 — Fission Plane in the Trophosome Region of P. cf polyhymnia. In vivo squeeze preparation of P. cf polyhymnia under incident light. (A) The animal exhibits a fission plane (arrowhead) in the trophosome region. The inset shows a higher magnification of the fission plane. Scale bar in the main figure 100 µm and in the inset 50 µm. (TIF) [file pone.0034709.s003.tif]

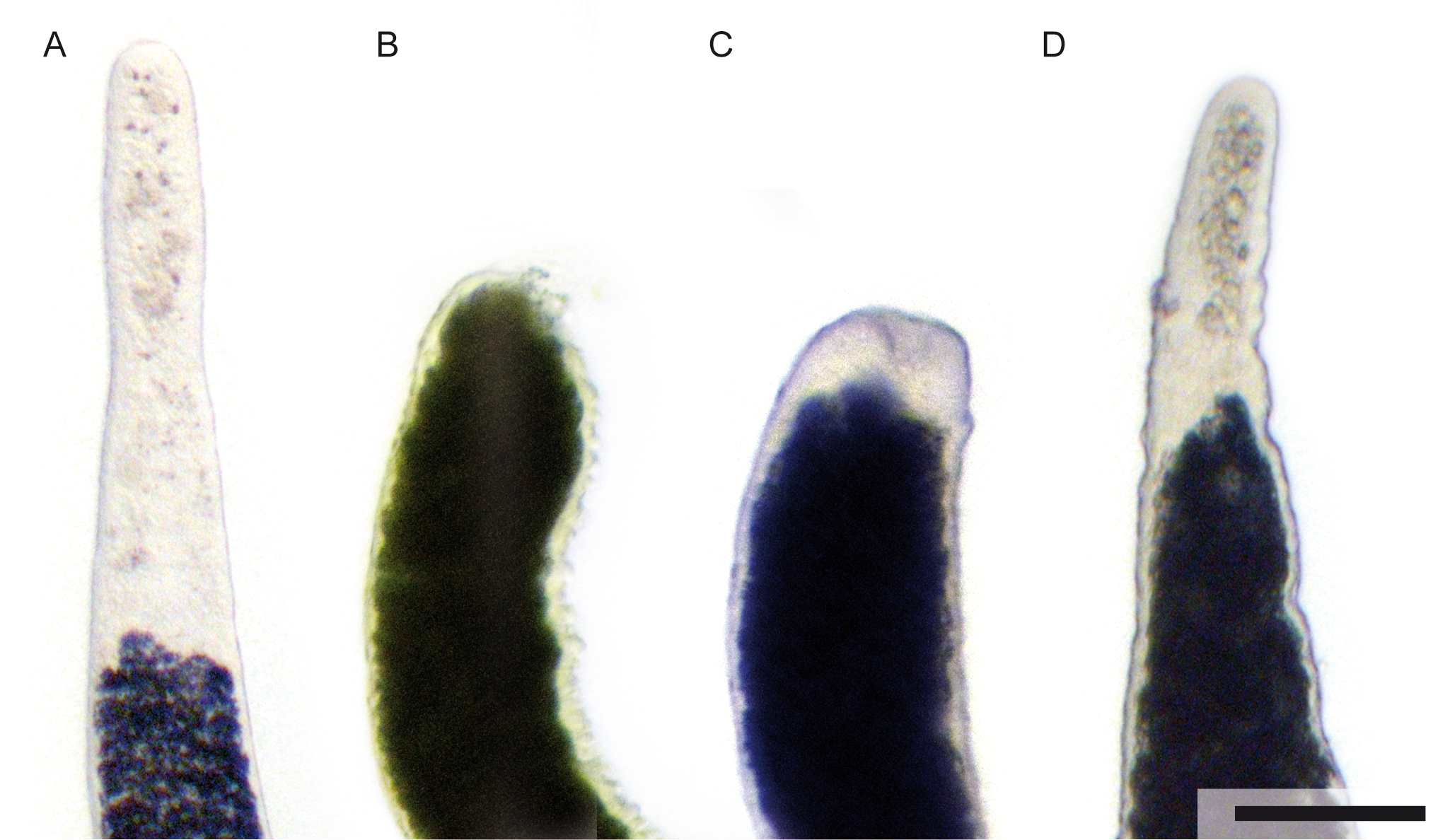

Supplement: Figure S4 — Regeneration After Rostrum Amputation of P. cf polyhymnia. Micrographs of regenerating P. cf polyhymnia. Live worms before (A) and directly after rostrum amputation (B). Rostrum regeneration 24 h (C) and 48 h (D) after amputation. Scale bar in (A–D) 50 µm. (TIF) [file pone.0034709.s004.tif]

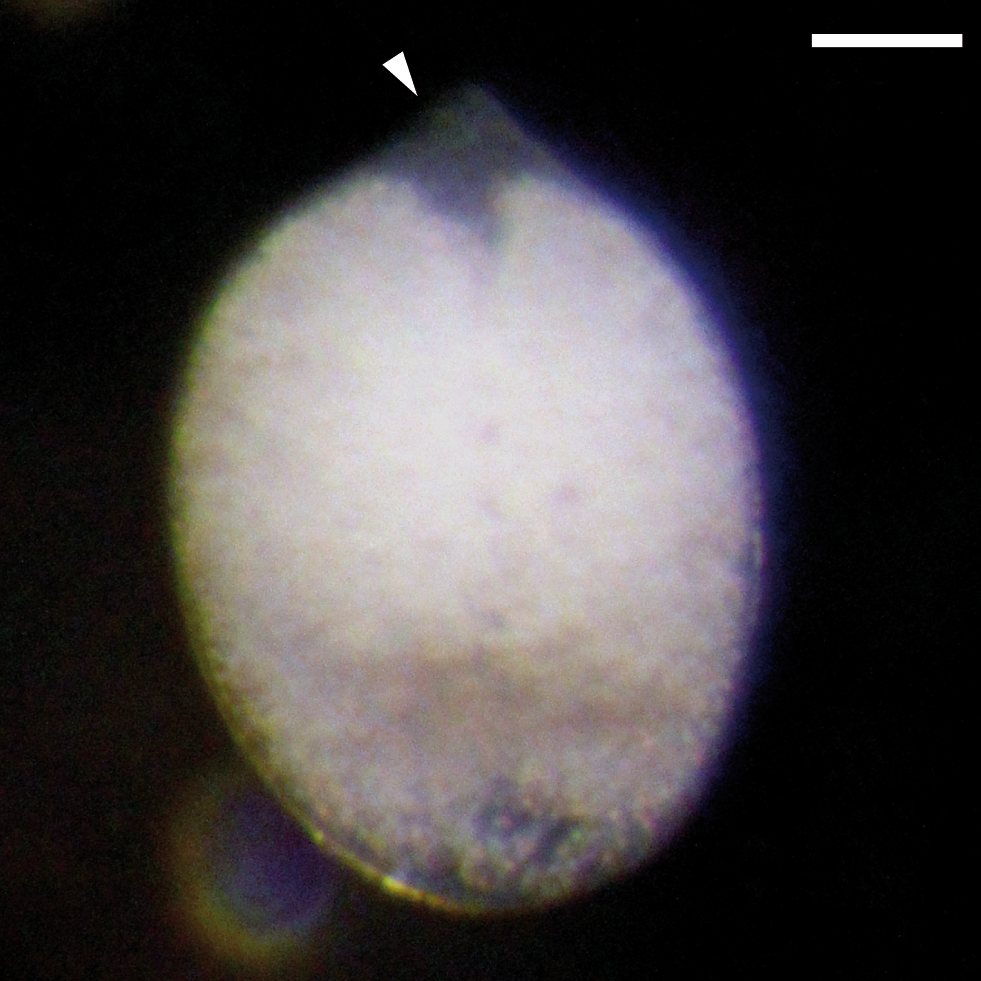

Supplement: Figure S5 — Regeneration of Tiny Trophosome Region Fragments of P. galateia. Micrograph of a 0.5-mm-long regenerating trophosome region fragment 14 days after rostrum amputation. A small rostrum is visible on one side (arrowhead). Scale bar 100 µm. (TIF) [file pone.0034709.s005.tif]
